# pH-Sensitive Micelles Based on Star Copolymer Ad-(PCL-*b*-PDEAEMA-*b*-PPEGMA)_4_ for Controlled Drug Delivery

**DOI:** 10.3390/polym10040443

**Published:** 2018-04-14

**Authors:** Huiyan Yang, Jianwei Guo, Rui Tong, Chufen Yang, Jem-Kun Chen

**Affiliations:** 1 School of Chemical Engineering & Light Industry, Guangdong University of Technology, Guangzhou 510006, China; huiyanyang1991@163.com (H.Y.); cfyang@gdut.edu.cn (C.Y.); 2 Guangzhou Tinci Materials Technology Co., Ltd., Guangzhou 510760, China; tongruisc@163.com; 3 Department of Materials Science and Engineering, National Taiwan University of Science and Technology, No. 43, Sec. 4, Keelung Road, Taipei 106, Taiwan

**Keywords:** anti-cancer drug delivery, polymeric micelles, pH-responsive, star polymer, adamantane

## Abstract

Enhancing drug loading efficacy and stability of polymeric micelles remains a grand challenge. Here we develop adamantane-based star copolymers adamantane-[poly(ε-caprolactone)-*b*-poly(2-(diethylamino)ethyl methacrylate)-*b*-poly(poly(ethylene glycol) methyl ether methacrylate)]_4_ (Ad-(PCL-*b*-PDEAEMA-*b*-PPEGMA)_4_) and their self-assembled micelles for controlled drug delivery. Results show that the polymers have excellent stability in solution with low critical micelle concentration (CMC) (0.0025–0.0034 mg/mL) and the apparent base dissociation constant (p*K*_b_) of the polymers is from 5.31 to 6.05. Dynamic light scattering analysis exhibits the great environmental response capability of the pH-sensitive micelles according to particle sizes and zeta potentials. With the synergy effect of the adamantane and hydrophobic block, the micelles display the high Doxorubicin (DOX) loading efficacy (up to 22.4%). The DOX release study shows that the micelles are capable of controlled release for drug. This work indicates the Ad-(PCL-*b*-PDEAEMA-*b*-PPEGMA)_4_ micelles may provide new guidelines for drug control and release system in overcoming cancer treatment.

## 1. Introduction

Currently, cancer is a major public health problem in the world [1,2], but the diagnosis and treatment of cancer are still a challenge in the field of human biomedicine [3,4,5]. Owing to non-specific distribution of drugs, conventional pharmacotherapy often kills normal cells and causes toxicity to the patient [6,7,8,9]. In addition, low concentrations of chemotherapy drugs in tumor tissue results in poor therapeutic efficacy [6,10]. Nanoparticle therapy shows unique advantages in pharmacotherapy, helping to improve the drug’s aqueous dispersity, toxicity profile, pharmacokinetic properties, as well as bioavailability. The pH of normal physiological tissue is about 7.4, the pH of extracellular solid tumors is 6.5–7.4, and the pH values of endosomes and lysosomes in tumor cells are 5.0 and 4.5–5.0, respectively [11,12]. With great difference in pH between human normal cells and a tumor cells’ microenvironment, there are tremendous potential applications of a pH-responsive drug delivery system for efficient drug delivery to the tumor.

Varieties of materials responding to biological stimuli have been designed to improve drug delivery systems [13,14]. In 2012, Zhang et al. [15] developed a series of pH-responsive block copolymers poly (ethylene glycol) methyl ether-*b*-(poly lactic acid-*co*-poly (β-amino esters)) (MPEG-*b*-(PLA-*co*-PAE)) through a Michael-type step polymerization. It was found that self-assembled micelles had a high drug loading capacity (18%) and great pH-responsive release performance. The release of drugs was significantly accelerated from micelles as the pH dropped from 7.4 to 5.0. However, the delivery system formed by linear amphiphilic block polymers is unstable in vivo environment as compared to star polymers. In 2013, Yang et al. [16] developed a series of 4-armed star triblock copolymers poly(ε-caprolactone)-*b*-poly(2-(diethylamino)ethyl methacrylate)-*b*-poly(poly(ethylene glycol) methyl ether methacrylate) (4As-PCL-PDEAEMA-PPEGMA) based on a tetrafunctional alcohol core. It was found that star copolymers had a low critical micelle concentration (2.2–4.0 mg/L) and pH-dependent properties. However, 40% of Doxorubicin (DOX) was released from micelles at pH 7.4, leading to unwanted side-effects on normal cells.

Although pH-sensitive polymeric micelles have the potential to revolution cancer diagnosis and therapy, there are significant challenges in translating basic research to clinical applications. A number of self-assembled micelles are unstable in vivo environment, and the interaction among blood components and carriers has been reported to induce drug leakage [17,18,19]. In addition, low drug loading capacity of existing carriers is another hurdle to their widespread application. The low drug loading content makes it necessary to use exogenous materials (for making carriers) frequently, which may induce significant safety problems [20]. Hence, it would therefore be desirable to develop a delivery system that possesses excellent stability and high drug loading capacity. The adamamtane with unique lipophilicity and biocompatibility are widely used in medicine, functional materials as well as nanotechnology [13,21,22]. Thus pH-sensitive polymeric micelles with adamamtane as the molecular core may provide a new guideline for drug control and release system in overcoming cancer treatment.

**Scheme 1:**
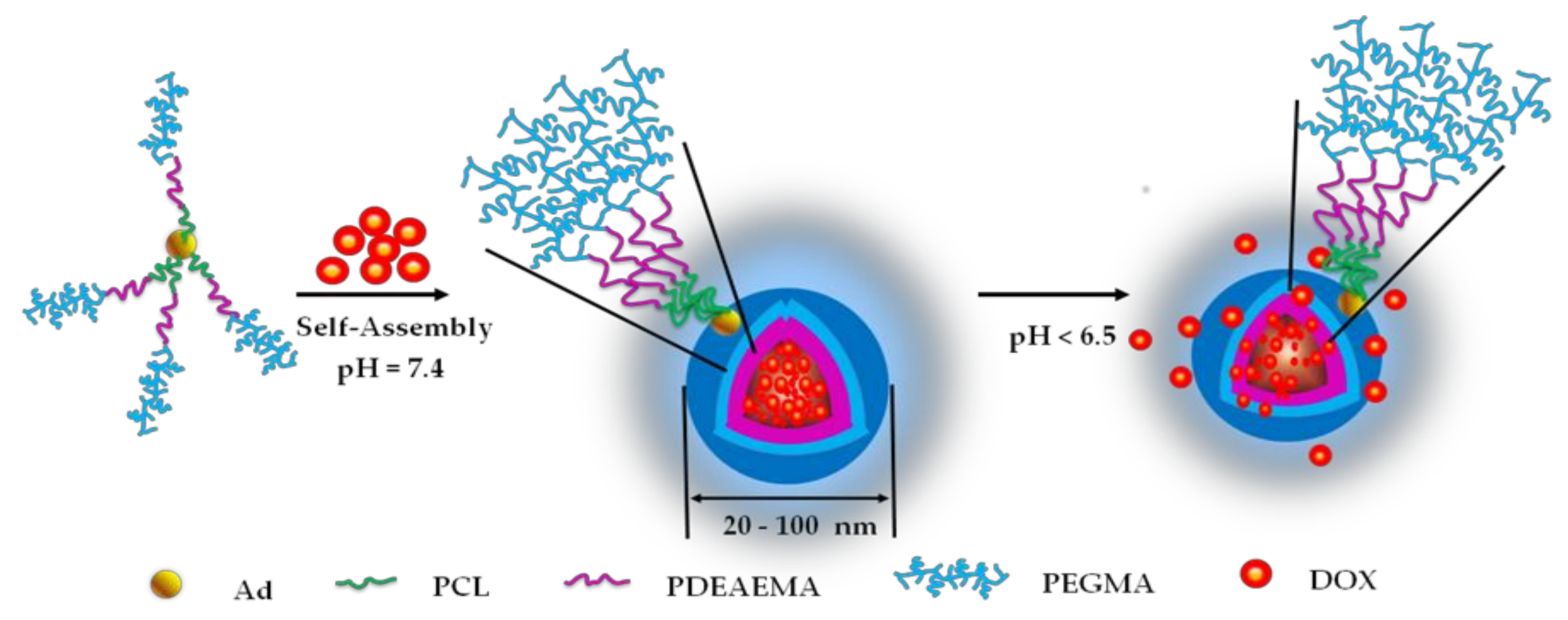
Illustration of the formation of the drug-loaded adamantane-[poly(ε-caprolactone)-*b*-poly(2-(diethylamino)ethyl methacrylate)-*b*-poly(poly(ethylene glycol) methyl ether methacrylate)]_4_ (Ad-(PCL-*b*-PDEAEMA-*b*-PPEGMA)_4_) micelles and the pH-dependent release of Doxorubicin (DOX).

In this work, we report a novel star amphiphilic polymer adamantane-[poly(ε-caprolactone)-*b*-poly(2-(diethylamino)ethyl methacrylate)-*b*-poly(poly(ethylene glycol) methyl ether methacrylate)]_4_ (Ad-(PCL-*b*-PDEAEMA-*b*-PPEGMA)_4_), which has a highly drug-loaded capacity and high stability of micelles featured by adamantane as the molecular core. In order to further study the relationship between the structure of polymer and the performance of micelles, a series of Ad-(PCL-*b*-PDEAEMA-*b*-PPEGMA)_4_ with different molecular weights are synthesized. During the synthesis, 1,3,5,7-tetrahydroxy adamantane (Ad-(OH)_4_) is used as a tetrafunctional initiator for ring-opening polymerization (ROP) of ε-caprolactone to obtain adamantane-[poly(ε-caprolactone)]_4_ [denoted as Ad-(PCL)_4_]. Ad-(PCL)_4_ is modified with 2-bromoisobutyryl bromide to prepare adamantane-[poly(ε-caprolactone)-bromine]_4_ [termed as Ad-(PCL-Br)_4_]. Ad-(PCL-Br)_4_ is employed as a macroinitiator for continuous activators regenerated by electron transfer atom transfer radical polymerization (ARGET ATRP) of 2-(Diethylamino) ethyl methacrylate and poly(ethylene glycol) methyl ether methacrylate to synthesize Ad-(PCL-*b*-PDEAEMA-*b*-PPEGMA)_4_. During the preparation of pH-responsive micelles, DOX is employed as a model, and the star polymers self-assemble by dialysis. A schematic of the encapsulation and release of DOX is shown in Scheme 1. DOX are expected to encapsulate into the micelles and keep stable in the normal physiological environment of pH = 7.4, and DOX are released from micelles in weakly acidic pH, which results from the protonation of amine groups of PDEAEMA. In this work, critical micelle concentration (CMC), p*K*_b_, particle size, zeta potential, loading content, entrapment efficiency, as well as drug release performance of micelles, are investigated in detail.

## 2. Materials and Methods

### 2.1. Materials

ε-caprolactone (ε-CL, Mackin, Shanghai, China) was stirred with CaH_2_ for 48 h, followed by distillation under reduced pressure. 2-(Diethylamino) ethyl methacrylate (DEAEMA, Energy Chemical, Shanghai, China) and poly(ethylene glycol) methyl ether methacrylate (PEGMA, *M*_n_ = 500 Da, Energy Chemical, Shanghai, China) were passed through a column of basic alumina to remove the inhibitors and stored at −18 °C. Triethylamine (TEA, Aldrich, Saint Louis, MO, USA) and tetrahydrofuran (THF, Energy Chemical, Shanghai, China) were stirred overnight with CaH_2_ and then distilled at atmospheric pressure. Toluene (Energy Chemical, Shanghai, China) was soaked with the activated 4Å molecular sieves. *N*,*N*,*N′*,*N′*,*N″*-pentamethyldiethylenetriamine (PMDETA) was purchased from Energy Chemical (Shanghai, China) and used as received. Stannous octoate (Sn(Oct)_2_), 2-Bromoisobutyryl bromide (BIBB), Cupric bromide (CuBr_2_) and pyrene were purchased from Shanghai Macklin Biochemical Co., Ltd. (Shanghai, China) and used as received. Doxorubicin hydrochloride (DOX·HCl) which was purchased from Beijing HuaFeng United Technology Co., Ltd. (Beijing, China), was dispersed in the phosphate buffer (pH 7.4), static for 24 h, and collected by centrifuging before being used. All other reagents were used as received without further purification.

### 2.2. Measurements

The chemical structure of the polymers was measured by Bruker AVANCE III 400 MHz superconducting fourier (Bruker, Billerica, MA, USA) at 25 °C using chloroform (CDCl_3_) as deuterated solvent. The number average molecular weight (*M*_n_) and dispersity index (*M*_w_/*M*_n_) of the polymers were characterized by gel permeation chromatography (GPC) with THF as the eluent (1.0 mL/min) and monodisperse polystyrene solution as standard. The GPC system was carried out with a Waters 1525/2414 system (Waters, Milford, MA, USA) equipped with isocratic high performance liquid chromatography (HPLC) pump and refractive index detector. A FluoroMax-4 fluorescence spectrometer (HORIBA Jobin Yvon, Clifton Park, NY, USA) was used to determine CMC of the star polymers using pyrene as the fluorescence probe. A fluorescence scanner ranging from 240 to 350 nm was performed with 373 nm emission wavelength and the scanning interval was 4 nm at room temperature. The diameter (*D*_h_) and zeta potential of blank micelles was examined via dynamic light scattering (DLS) by a ZetaPALS zeta potential and granularity analyzer (Brookhaven, New York, NY, USA), and the studied samples were filtered through a 0.45 m filter before testing. A TalosF200S transmission electron microscopy (TEM, FEI, Hillsboro, OR, USA) was employed to characterize the morphologies of drug-loaded micelles.

### 2.3. Synthesis of Ad-(PCL-b-PDEAEMA-b-PPEGMA)_4_ Copolymers

Three star polymers Ad-(PCL-*b*-PDEAEMA-*b*-PPEGMA)_4_ with different feed ratios were synthesized by comprehensively utilizing the technology of adamantane chemistry [22], ROP and ARGET ATRP. Synthetic procedure of Ad-(PCL-*b*-PDEAEMA-*b*-PPEGMA)_4_ is as follows. Notably, Ad-(OH)_4_, a four-directional initiator for ROP of ε-CL was prepared according to the reported procedure with adamantane as the starting material [23].

#### 2.3.1. Synthesis of Ad-(PCL)_4_

Ad-(PCL)_4_ was prepared by the ROP of ε-CL in the presence of Ad-(OH)_4_ as initiator and Sn(Oct)_2_ as the catalyst [24]. Ad-(OH)_4_ (0.126 g, 0.63 mmol) was added into a 100 mL dried two-necked flask with magnetic stir bar, and the flask was then evacuated and purged with argon (Ar) three times. Under the protection of Ar, ε-CL (6.32 g) and Sn(Oct)_2_ (0.1% wt of ε-CL, 0.008 g) solution in toluene were added into the flask in turn by syringe, followed by disperse ultrasonic treatment for 10 min. Subsequently, the reaction apparatus was transferred to a thermostat controlled oil bath at 120 °C for 24 h. Later, the toluene was removed by rotary evaporation, and the crude product was dissolved in THF, followed by being dropwisely added into 10-fold excess of *n*-hexane. The resulting product was collected by suction filtration and dried at 40 °C in vacuum oven for 24 h.

#### 2.3.2. Synthesis of Ad-(PCL-Br)_4_

Ad-(PCL-Br)_4_, a macroinitiator, was synthesized by adding Ad-(PCL)_4_ (3 g, 0.256 mmol) to a dried flask equipped with a magnetic stirring bar under argon, and anhydrous THF (20 mL) and TEA (0.71 mL, 5.11 mmol) was added into the flask in turn. BIBB (0.60 mL, 5.11 mmol) in anhydrous THF (5 mL) was added dropwisely into the flask with vigorous stirring under ice bath conditions. The reaction was cooled down to room temperature and stirred for a period of 24 h. Subsequently, the quaternary ammonium salt produced by the reaction was removed by a neutral alumina column and then most of the THF was evaporated by rotary evaporation. The resulting solution was added dropwisely into cold *n*-hexane and the precipitate was separated by filtration and dried at 40 °C in vacuum oven for 24 h.

#### 2.3.3. Synthesis of Ad-(PCL-*b*-PDEAEMA-*b*-PPEGMA)_4_

Ad-(PCL-*b*-PDEAEMA-*b*-PPEGMA)_4_ was synthesized via ARGET ATRP [25] using Ad-(PCL-Br)_4_ as the macroinitiator. Ad-(PCL-Br)_4_ (1.5 g, 0.12 mmol) and the catalyst CuBr_2_ (0.027 g, 0. 12 mmol) were added to a 100 mL dried Schlenk flask with a magnetic stirrer under argon. Solvent anhydrous THF (18 mL), monomer DEAEMA (1.6 g, 8.64 mmol), ligand PMDETA (0.05 mL, 0.24 mmol) were sequentially added to the reaction flask with syringes. After stirring for 10 min to promote the formation of catalyst complex CuBr_2_/PMDETA, the mixed solution was transferred to an oil bath at 65 °C. Subsequently, reducing agent Sn(Oct)_2_ (0.097 g, 0.24 mmol) in THF (2 mL) was added into the flask by syringe. After 4 h, monomer PEGMA (1.92 g, 3.84 mmol) was added to carry out continuous polymerization. After the reaction, the mixed solution was cooled down to room temperature, and diluted with THF. CuBr_2_ was removed by flowing through a neutral alumina column, and the solution was concentrated on a rotary evaporator. The resulting solution was added dropwisely to n-hexane to obtain precipitate which was filtered and dried in vacuum oven at 40 °C for 24 h to obtain the final polymer.

#### 2.3.4. Determination of CMC Values

The formation of polymeric micelles self-assembled from Ad-(PCL-*b*-PDEAEMA-*b*-PPEGMA)_4_ in aqueous solution was monitored on a fluorescence spectrometer with a pyrene as the probe. The polymer was dissolved in acetone, followed by diluted with deionized water to obtain a polymer mother liquor at the concentration of 0.1 mg/mL, which was stirred for 24 h to remove acetone. Sequentially, the polymer solution was diluted to a series of concentration from 0.0001 to 0.1 mg/mL and then combined with a pyrene solution (12 × 10^−7^ M) to obtain polymer/pyrene solutions. The mixed solution was equilibrated at room temperature in the dark for 48 h before test. Notably, the concentration of pyrene was controlled at 6 × 10^−7^ M. The fluorescence excitation spectra of the polymer/pyrene solutions were employed to characterize the CMC value of Ad-(PCL-*b*-PDEAEMA-*b*-PPEGMA)_4_. Each experimental point was done in triple.

#### 2.3.5. Acid–Base Titration

The Ad-(PCL-*b*-PDEAEMA-*b*-PPEGMA)_4_ polymers and NaCl (control) were dissolved in deionized water (1 mg/mL) and the initial pH value of the solution was adjusted to 3 with 0.2 M HCl. 0.1 M NaOH was added stepwisely into the resulting solution while the pH of the solution was measured with a pH meter. All titrations were repeated three times.

#### 2.3.6. Micelle Preparation and Characterization

The self-assembled polymeric micelles were prepared by dialysis method. Briefly, 5 mg polymer Ad-(PCL-*b*-PDEAEMA-*b*-PPEGMA)_4_ was dissolved in 20 mL of DMSO, followed by adding dropwisely 50 mL of phosphate buffer (pH 7.4). The mixed solution was transferred into a dialysis bag (molecular weight cutoff, MwCO = 3.5 kDa) and then dialyzed against phosphate buffer (pH 7.4). Notably, the dialysate was refreshed every 2 h at the first 12 h and then every 6 h for 36 h. The resulting solution was filtered through a 0.45 μm filter to remove the large aggregates. The pH value of micellar solution was adjusted from 2.0 to 10.0 with NaOH or HCl (0.1 M). The particle sizes and zeta potentials of Ad-(PCL-*b*-PDEAEMA-*b*-PPEGMA)_4_ micelles at different pH were characterized by DLS analysis using a ZetaPALS zeta potential and granularity analyzer (Brookhaven, New York, NY, USA). Each experimental point was measured in triple.

#### 2.3.7. Encapsulation and Release of DOX

DOX was utilized as a model drug to test the properties of the polymer micelles as drug carriers. The self-assembled DOX-loaded Ad-(PCL-*b*-PDEAEMA-*b*-PPEGMA)_4_ micelles were prepared by dialysis technique. The polymer Ad-(PCL-*b*-PDEAEMA-*b*-PPEGMA)_4_ (40 mg) was dissolved in 20 mL of DMSO, and the DOX (10 or 20 mg) was added to the solution with vigorous stirring for 1 h, followed by dropwisely adding 40 mL of phosphate buffer (pH 7.4). The mixed solution was transferred into a dialysis bag (MwCO = 3.5 kDa) and then dialyzed against phosphate buffer (pH 7.4) at first 5 h and later against deionized water. After 48 h, the solution was filtered through a 0.45 μm filter and freeze-dried for characterization.

The drug loading content (DLC) and entrapment efficiency (EE) of micelles was evaluated in 0.1 mg/mL micellar solution on a UV–Vis spectrophotometer (UV-9600) (Shanghai, China) by monitoring the absorbance at 480 nm. DLC and EE were calculated as follows:

(1) $$\mathrm{DLC}=\frac{\mathrm{weight}\;\mathrm{of}\;\mathrm{loaded}\;\mathrm{drug}}{\mathrm{weight}\;\mathrm{of}\;\mathrm{polymer}\;\mathrm{and}\;\mathrm{loaded}\;\mathrm{drug}}\times 100\%$$

(2) $$\mathrm{EE}=\frac{\mathrm{weight}\;\mathrm{of}\;\mathrm{loaded}\;\mathrm{drug}}{\mathrm{weight}\;\mathrm{of}\;\mathrm{drug}\;\mathrm{in}\;\mathrm{feed}}\times 100\%$$

The DOX release properties from drug–loaded Ad-(PCL-*b*-PDEAEMA-*b*-PPEGMA)_4_ micelles were investigated in phosphate buffer or acetic buffer (DOX/polymer = 20/40). Typically, 5 mg of lyophilized DOX-loaded micelles was dispersed in 5 mL of phosphate buffer (pH 7.4, 6.8, 6.5, 6.0 ) or acetic buffer (pH 5.5, 5.0, 4.5) and then transferred into a dialysis bag (MwCO = 3.5 kDa), followed by stirring in a shaking water bath at 37 °C and 100 rpm. Along with taking out 4 mL of solution at a certain time interval, the same volume of fresh buffer was immediately added to keep the total volume of the solution. The concentration of DOX was monitored at 480 nm by UV–Vis spectrophotometer. The cumulative release of DOX was calculated by the following formula. Each experiment was repeated three times.

(3) $$E_{r}=\frac{V_{e}\sum_{1}^{n-1}c_{i}+c_{0}V_{0}}{m_{DOX}}\times 100\%$$

where *V_e_* = 4 mL; *V*_0_ = 44 mL; *c_i_* is the sample concentration at the *i-*th replacement sample; *m_DOX_* represents the amount of DOX in the micelles.

## 3. Results and Discussion

### 3.1. Synthesis and Characterization of Ad-(PCL-b-PDEAEMA-b-PPEGMA)_4_ Star Copolymers

**Scheme 2:**
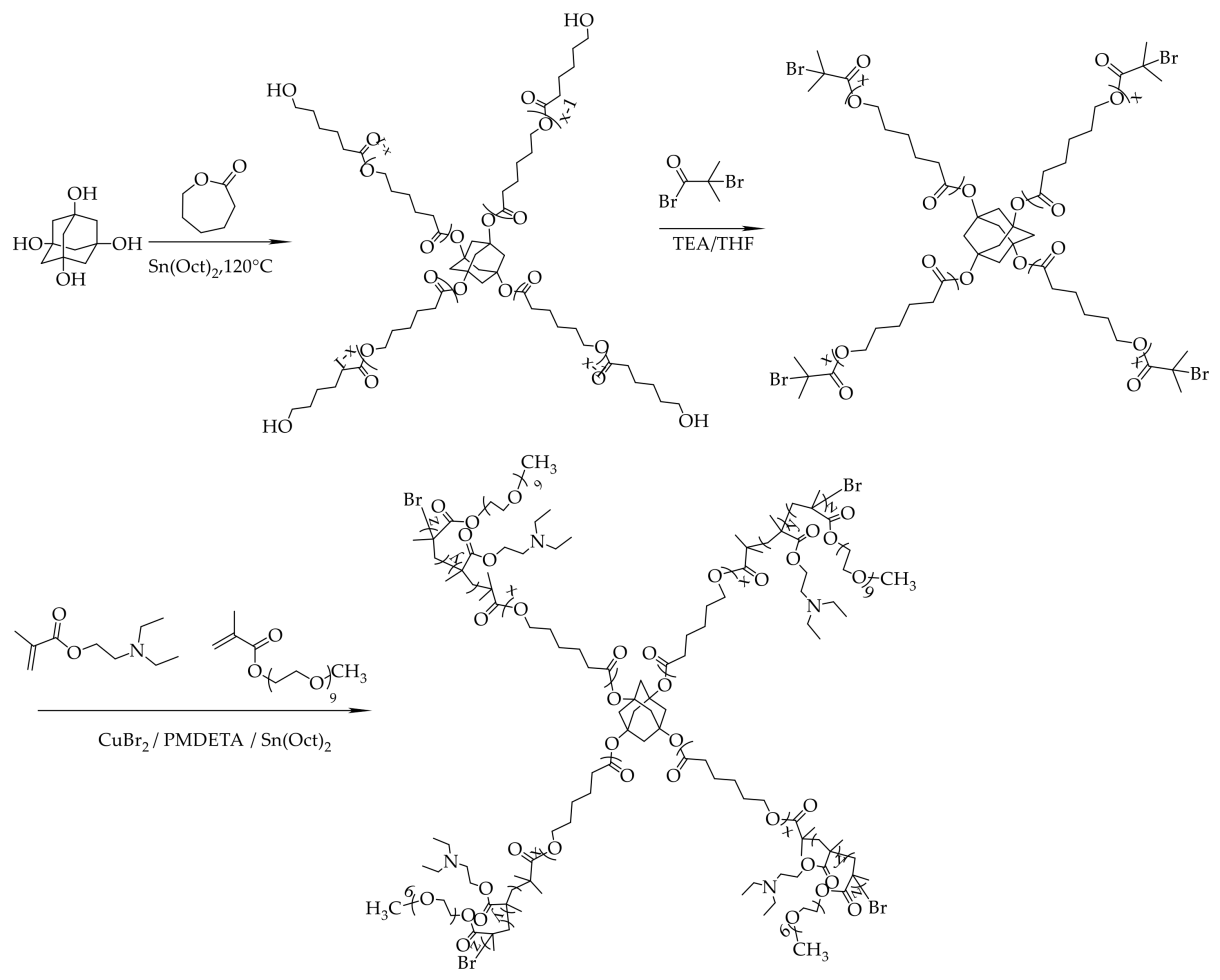
Synthetic Route of Star Copolymer Ad-(PCL-*b*-PDEAEMA-*b*-PPEGMA)_4_.

Ad-(PCL-*b*-PDEAEMA-*b*-PPEGMA)_4_ is prepared according to a combination of ROP and ARGET ATRP, as shown in Scheme 2. Firstly, Ad-(PCL)_4_ is synthesized by ROP of ε-CL with Ad-(OH)_4_ as initiator. Secondly, the synthesis of Ad-(PCL-Br)_4_ is carried out by using Ad-(PCL)_4_ with 5 equivalent BIBB. Finally, Ad-(PCL-*b*-PDEAEMA-*b*-PPEGMA)_4_ is polymerized in THF at 65 °C by using Ad-(PCL-Br)_4_ as macroinitiator, and the mole ratio of Ad-(PCL-Br)_4_/PMDETA/CuBr_2_/Sn(Oct)_2_ is 1:2:1:2.

**Figure 1:**
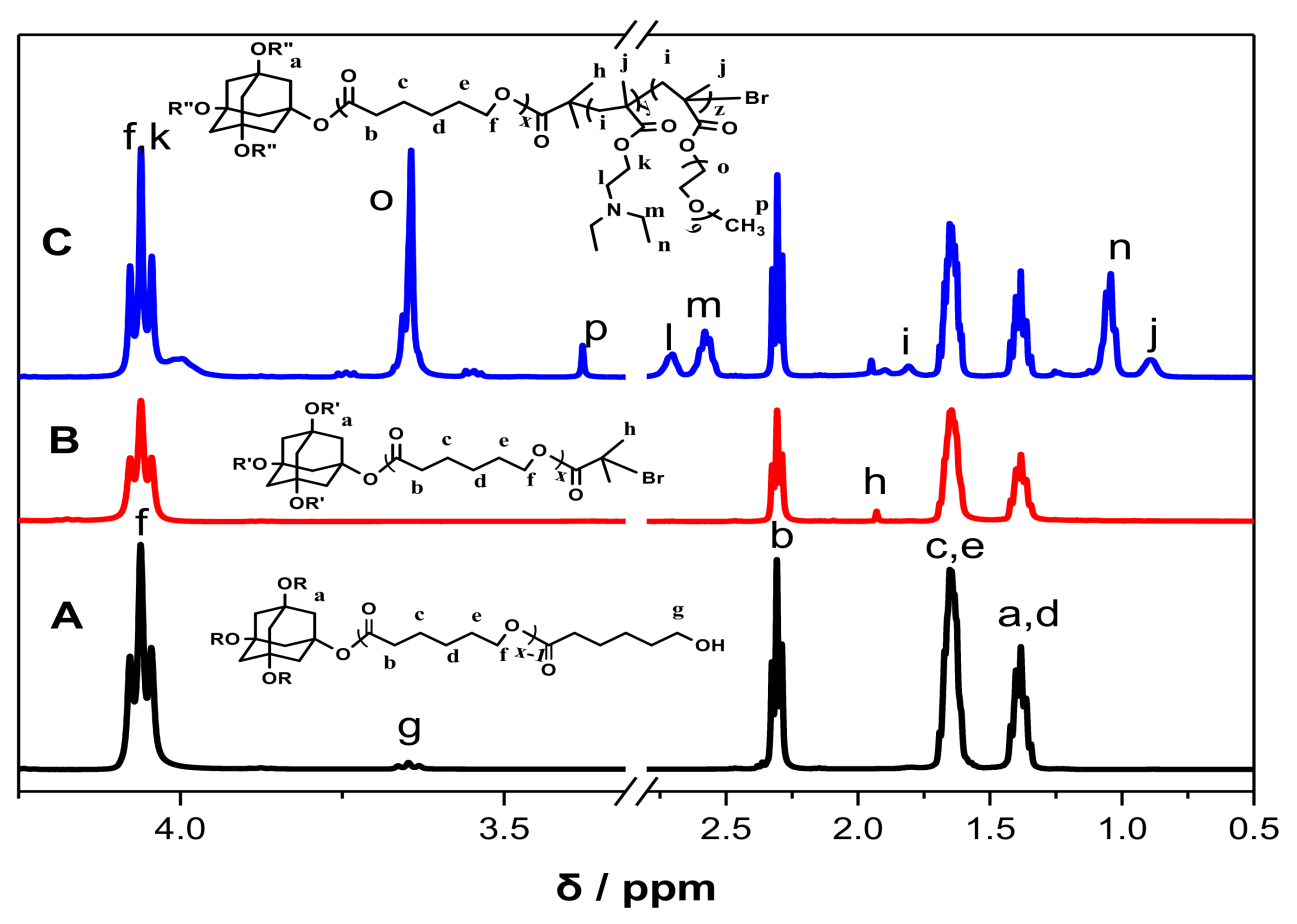
^1^H NMR spectra of Ad-(PCL)_4_ (**A**), Ad-(PCL-Br)_4_ (**B**) and Ad-(PCL-*b*-PDEAEMA-*b*-PPEGMA)_4_ (**C**).

By changing the feed ratio, three amphiphilic polymers of Ad-(PC_22_-*b*-PD_18_-*b*-PP_8_)_4_ Ad-(PC_28_-*b*-PD_18_-*b*-PP_8_)_4_ and Ad-(PC_28_-*b*-PD_25_-*b*-PP_8_)_4_ were prepared. ^1^H NMR spectrum was employed to characterize the chemical structures and compositions of star polymers (Figure 1). The ^1^H NMR spectra of Ad-(PCL)_4_ (Figure 1A) shows that there are four major peaks: the triplet peak at 4.06 ppm is the characteristic resonances of the (–C**H**_2_–O–) protons, the triplet peak at 2.31 ppm belongs to the (–C**H**_2_–CO–) protons, the multiple peak at 1.65 ppm is ascribe to the (–O–CH_2_–C**H**_2_–CH_2_–C**H**_2_–CH_2_–CO–) protons, the multiple peak at 1.38 ppm is the characteristic resonances of the (–CH_2_–C**H**_2_–CH_2_–) protons, the tiny triplet peak at 3.65 ppm belongs to the (–C**H**_2_–OH) protons which are at the end of chains and the peak of the adamantane protons coincides with the peaks of (–CH_2_–C**H**_2_–CH_2_–) protons.

The ^1^H NMR spectrum of Ad-(PCL-Br)_4_ can be seen in Figure 1B. The peaks at 3.65 ppm disappears completely while the signals at 1.93 ppm appears, which are ascribe to (–C(C**H**_3_)_2_–Br) protons. This indicated that Ad-(PCL-Br)_4_ is prepared successfully.

The ^1^H NMR spectrum of Ad-(PCL-*b*-PDEAEMA-*b*-PPEGMA)_4_ is shown in Figure 1C. In addition to the peaks from Ad-(PCL-Br)_4_, the peaks at 0.89 and 1.81 ppm are assigned as (–CC**H**_3_) and (–C**H**_2_–) protons of the methacrylate backbone respectively; the peaks at 2.70 and 4.06 ppm are assigned as (–C**H**_2_C**H**_2_–) protons of DEAEMA unit; the peaks at 1.04 and 2.58 ppm are assigned as (–C**H**_2_C**H**_3_) protons of DEAEMA unit; the peaks at 3.38 and 3.64 ppm are the characteristic resonances of (–OC**H**_3_) and (–OC**H**_2_–C**H**_2_O–) protons of PEGMA unit, respectively.

**Figure 2:**
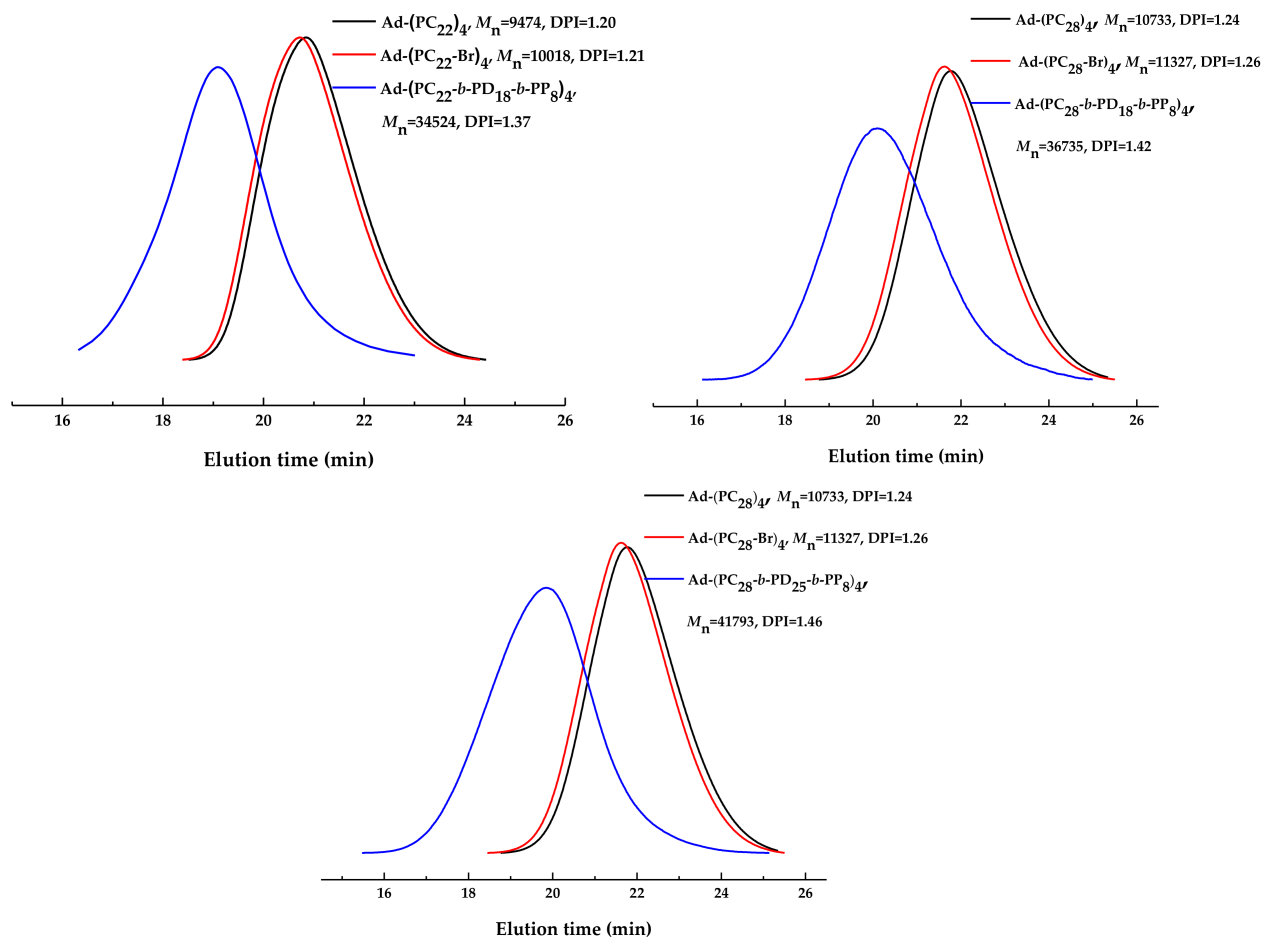
GPC traces of Ad-(PC_22_-*b*-PD_18_-*b*-PP_8_)_4_, Ad-(PC_28_-*b*-PD_18_-*b*-PP_8_)_4_, Ad-(PC_28_-*b*-PD_25_-*b*-PP_8_)_4_ and their intermediates.

The molecular weights (*M*_n_) and distribution coefficients (*M*_w_/*M*_n_) of the star polymers were characterized by GPC analysis (Table 1 and Figure 2). It can be seen that the polymers had a narrow molecular weight distribution (*M*_w_/*M*_n_ < 1.5) and the GPC trace curves of star copolymers and their intermediates in Figure 2 exhibit a symmetrical unimodal distribution, indicating that the ROP as well as ARGET ATRP process are controllable. However, the molecular weight measured by GPC is smaller than that measured by ^1^H NMR spectrum. The reason is that GPC uses linear polystyrene as a standard sample, and the hydrodynamic volume of star polymers is smaller than that of linear polymers [26,27].

### 3.2. CMC Values Determined by Fuorescence Analysis

**Figure 3:**
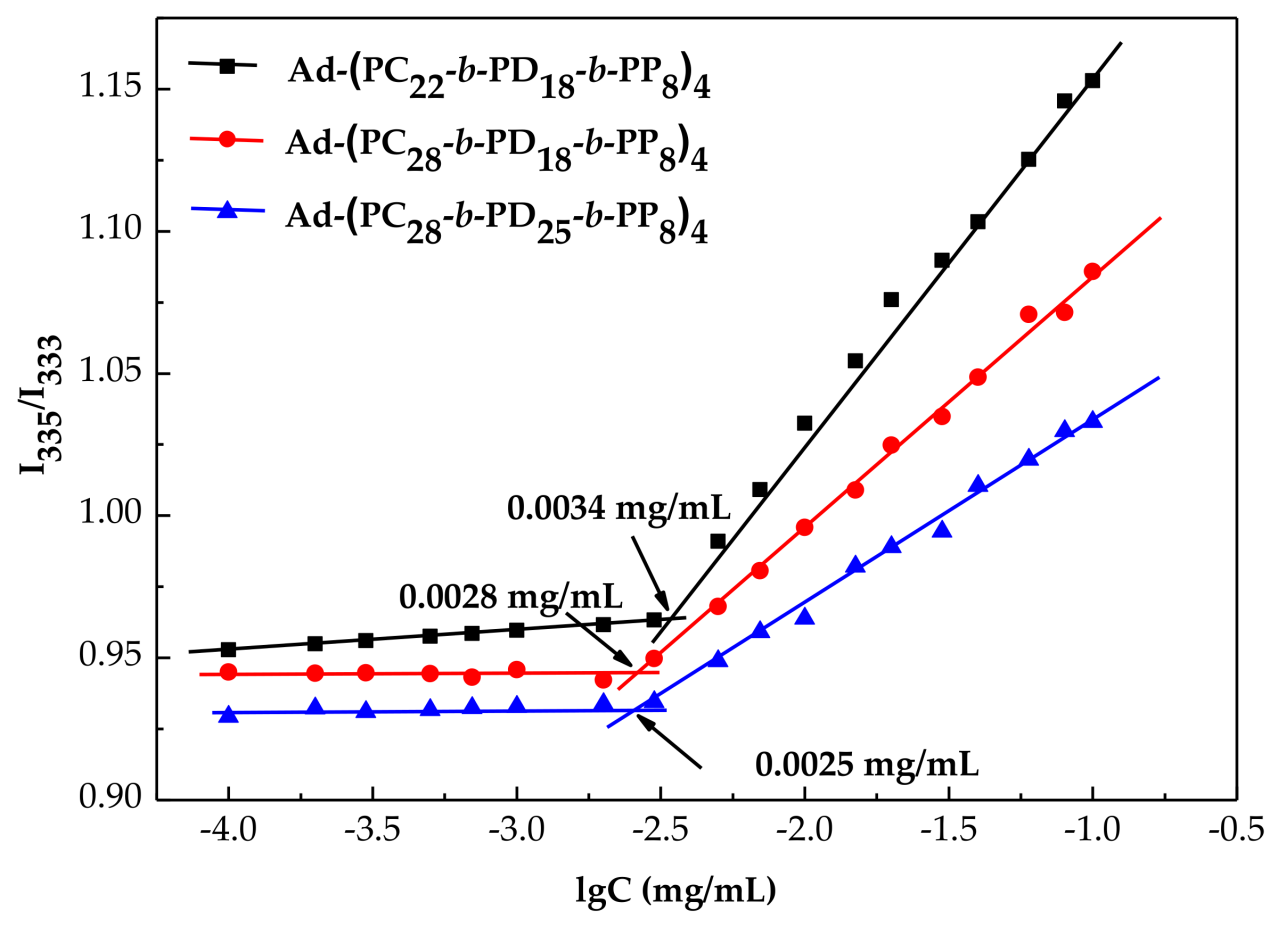
Graphs of intensity ratios (*I_335_*/*I_333_*) as function of logarithm of polymeric concentrations in aqueous solution.

It is generally believed that amphiphilic polymer with low CMC value would keep micelles stable in blood circulation system and prevent chemotherapy drugs release before reaching the cancer cells [28]. The CMC of amphiphilic polymers Ad-(PCL-*b*-PDEAEMA-*b*-PPEGMA)_4_ was performed on fluorescence spectroscopy in the presence of pyrene as the probe (Figure 3). As the concentration of Ad-(PCL-*b*-PDEAEMA-*b*-PPEGMA)_4_ increases, excitation spectrum intensity of pyrene increases and the third peak shifted from 333 to 335 nm. The CMC of copolymers is determined from the threshold concentration, where the intensity ratio *I*_335_/*I*_333_ begins to increase obviously. The CMC values of Ad-(PC_22_-*b*-PD_18_-*b*-PP_8_)_4_, Ad-(PC_28_-*b*-PD_18_-*b*-PP_8_)_4_ and Ad-(PC_28_-*b*-PD_25_-*b*-PP_8_)_4_ are 0.0034 mg/mL, 0.0028 mg/mL and 0.0025 mg/mL respectively. These values are lower than the CMC reported for common surfactant, indicating a long-circulating characteristics of the polymeric micelles. It should be noted that the longer the length of the PCL and PDEAEMA segment, the lower the CMC value. The reason is that the hydrophobic interactions of the copolymer is related to the length of the hydrophobic segment. An excellent lipophilicity and rigidity characteristics of adamantane and hydrophobicity of PDEAEMA in neutral conditions would be good to enhance the hydrophobicity of the polymers.

### 3.3. Titration of Ad-(PCL-b-PDEAEMA-b-PPEGMA)_4_ Copolymers

**Figure 4:**
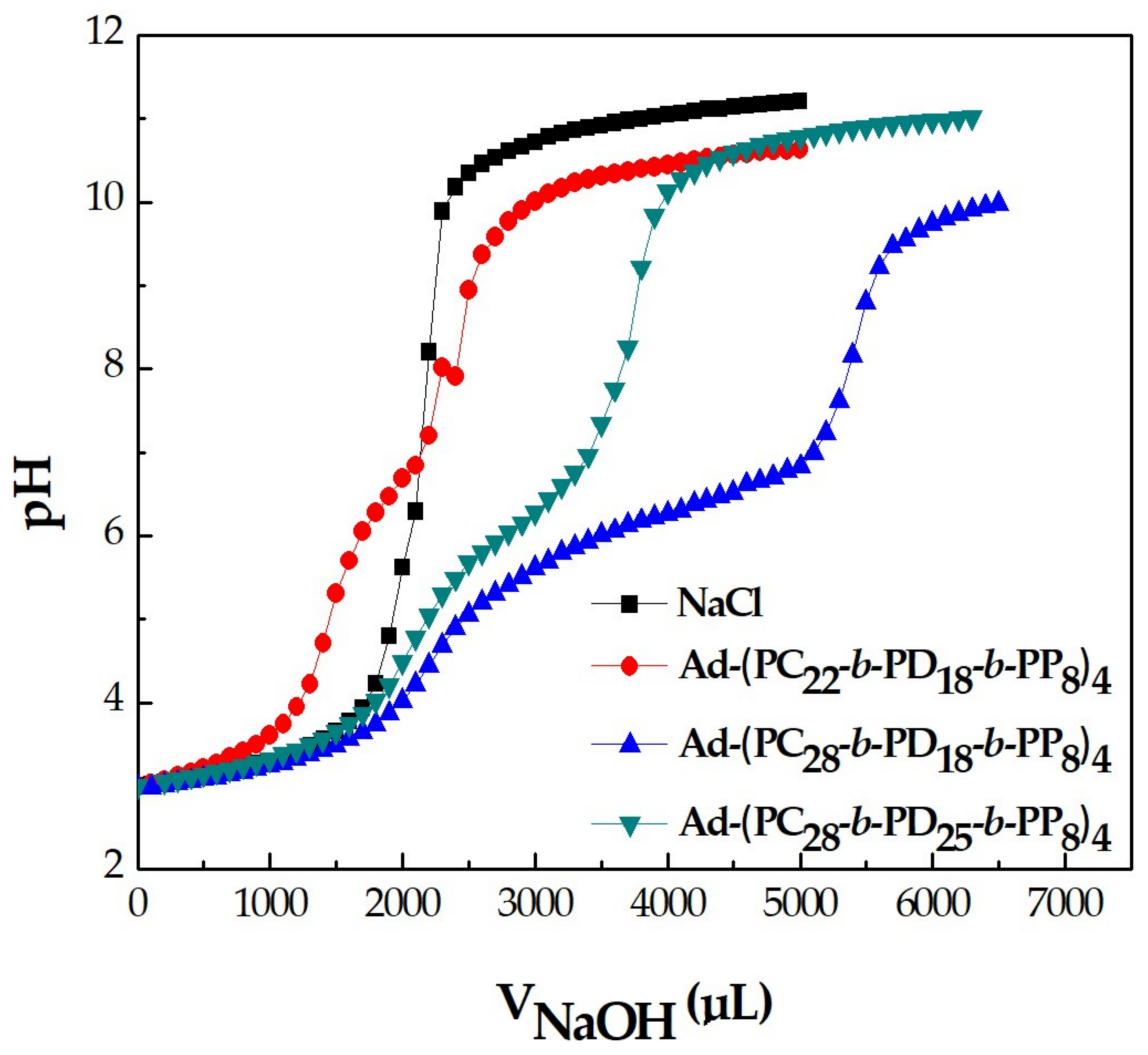
The pH-profile of Ad-(PCL-*b*-PDEAEMA-*b*-PPEGMA)_4_ (1 mg/mL)and NaCl (1 mg/mL)by acid–base titration with 0.2 M HCl and 0.1 M NaOH.

As shown in acid-base titration curve (Figure 4), the apparent p*K*_b_ of Ad-(PC_22_-*b*-PD_18_-*b*-PP_8_)_4_, Ad-(PC_28_-*b*-PD_18_-*b*-PP_8_)_4_ and Ad-(PC_28_-*b*-PD_25_-*b*-PP_8_)_4_ are 6.05, 5.68 and 5.31, respectively. The pH buffering region of polymers are in pH 4.22–7.20, 4.49–6.96, and 4.71–7.00. The results show potential of micelles in avoiding undesirable side-effects for normal cells.

### 3.4. Particle Size and Zeta Potential of the Micelles at Different pH

**Figure 5:**
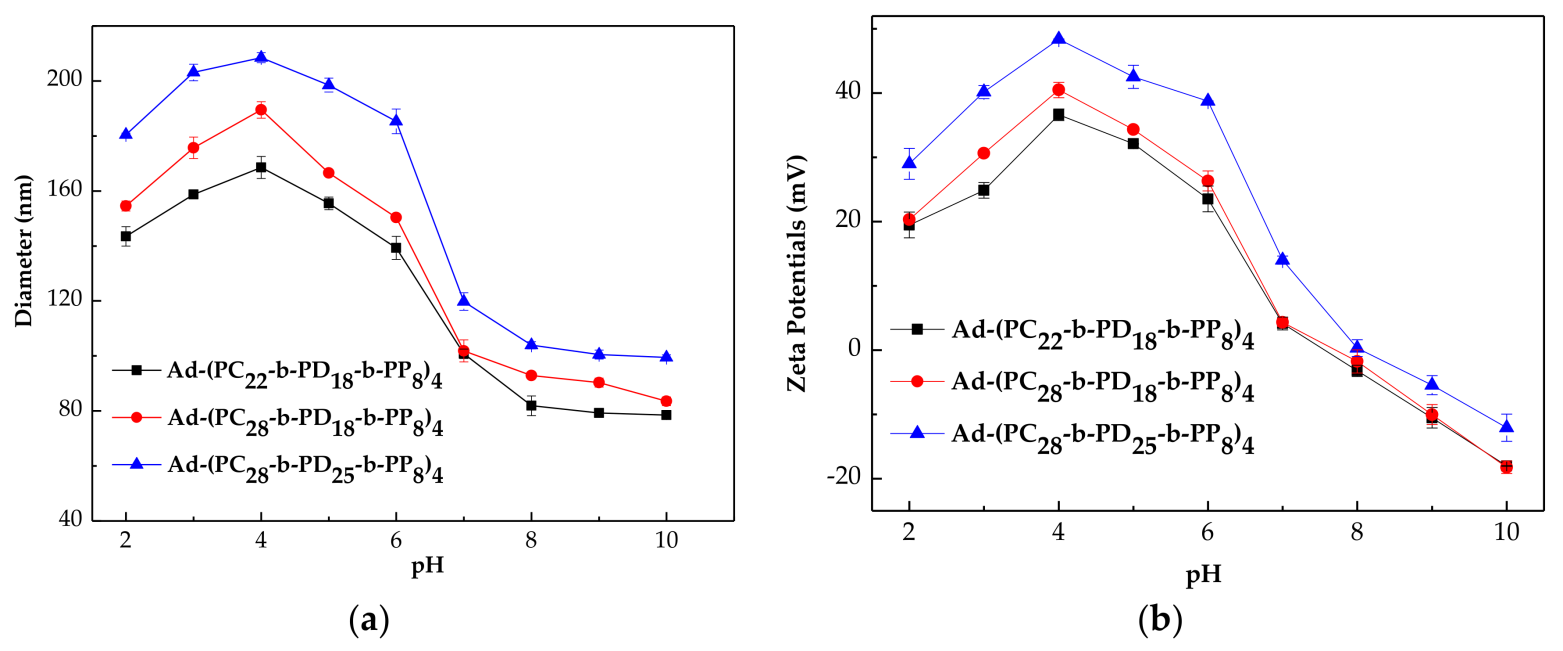
Effects of pH on the *D*_h_ (**a**) and zeta potential (**b**) of star polymeric micelles.

Figure 5a presents the effective diameter of micelles at different pH ranging from 2 to 10, which demonstrates pH-responsive behavior of self-assembled micelles. When pH value drops from 10 to 7, there is no significantly effect on the *D*_h_ of the micelles. The reason is that the tertiary amine groups of the PDEAEMA segment are completely deprotonated and PDEAEMA simultaneously forms the core of the micelles with the PCL segment. The reason why the *D*_h_ of micelles increases significantly when pH gradually decreases from 7 to 4 is that the tertiary amine groups of the PDEAEMA segment are protonated gradually and the micelles swell to balance the increasing electrostatic repulsions [29]. When pH drops to less than 4, the *D*_h_ of micelles decreases, which is attributed to the fact that the electrostatic repulsion is larger than intra-micellar hydrophobic interactions, thus the aggregation number of the polymers decreases.

Figure 5b presents the zeta potential of micelles as the pH decreases from 10 to 2. At pH > 8, the charge of the micelles is negative, which may attributed to the hydrolysis of ester-groups in basic medium. Further decreasing the pH of the micellar solution from 8 to 5 results in a increase in the zeta potential, which reflects the ongoing protonation process of the tertiary amine groups of PDEAEMA. In addition, the charge of the micelles is positive, which would enhance permeability and retention effect (EPR) of micelles for longer duration [30]. When the pH value drops from 4 to 2, the zeta potentials decrease slightly, which may result from the decrease of the aggregation number or the dissociation of the micelles [25]. These properties of micelles would trigger the target-specific delivery of drugs. Comparing the particle sizes and zeta potential of the micelles with different component mass ratios, the higher DEAEMA content, the better pH-responsiveness.

### 3.5. Encapsulation and pH-Triggered Release of DOX∙HCl

As shown in Table 2, the longer the hydrophobic segment, the higher drug loading content and entrapment efficiency, which indicates that loading content and entrapment efficiency of the DOX-loaded Ad-(PCL-*b*-PDEAEMA-*b*-PPEGMA)_4_ micelles are dependent on the content of PCL and PDEAEMA [31]. The reason is that the interaction between polymer chains and drugs is improved by enhancing the content of hydrophobic segment [32]. For example, Ad-(PC_22_-*b*-PD_18_-*b*-PP_8_)_4_ has an EE of 37.8% and DLC of 7.7%, Ad-(PC_28_-*b*-PD_18_-*b*-PP_8_)_4_ has an EE of 40.4% and DLC of 9.9%, while Ad-(PC_28_-*b*-PD_25_-*b*-PP_8_)_4_ has an EE of 56.0% and DLC of 11.6%. In addition, the drug loading capacity of micelles is increased by increasing the feeding concentration of DOX. Yang et al. once prepared the micelles by self-assembling from 4As-PCL-PDEAEMA-PPEGMA [16]. However, the highest DLC of micelles only reached 20.6%. By replacing the flexible core of pentaerythritol with the rigid adamantane, the DLC is up to 22.4%, which may be result from large stereo obstacles of adamantane. Moreover, the particle size of the DOX-loaded Ad-(PCL-*b*-PDEAEMA-*b*-PPEGMA)_4_ micelles increases after incorporation of DOX (feed ratios of DOX to polymer are 10–20 mg to 40 mg), which is about 110–256 nm. Presumably, the DOX boosts the hydrophobic interaction between the PCL, PDEAEMA and DOX, which results in the increase of the aggregation number. And the longer hydrophobic segment induces obvious larger sizes of micelles. However, the size of DOX-loaded micelles are larger than the theoretical values of the unimers, which might be attributed to the fact that DOX-loaded micelles are complex multimolecular micelles rather than unimolecular micelles at a concentration above CMC [33].

**Figure 6:**
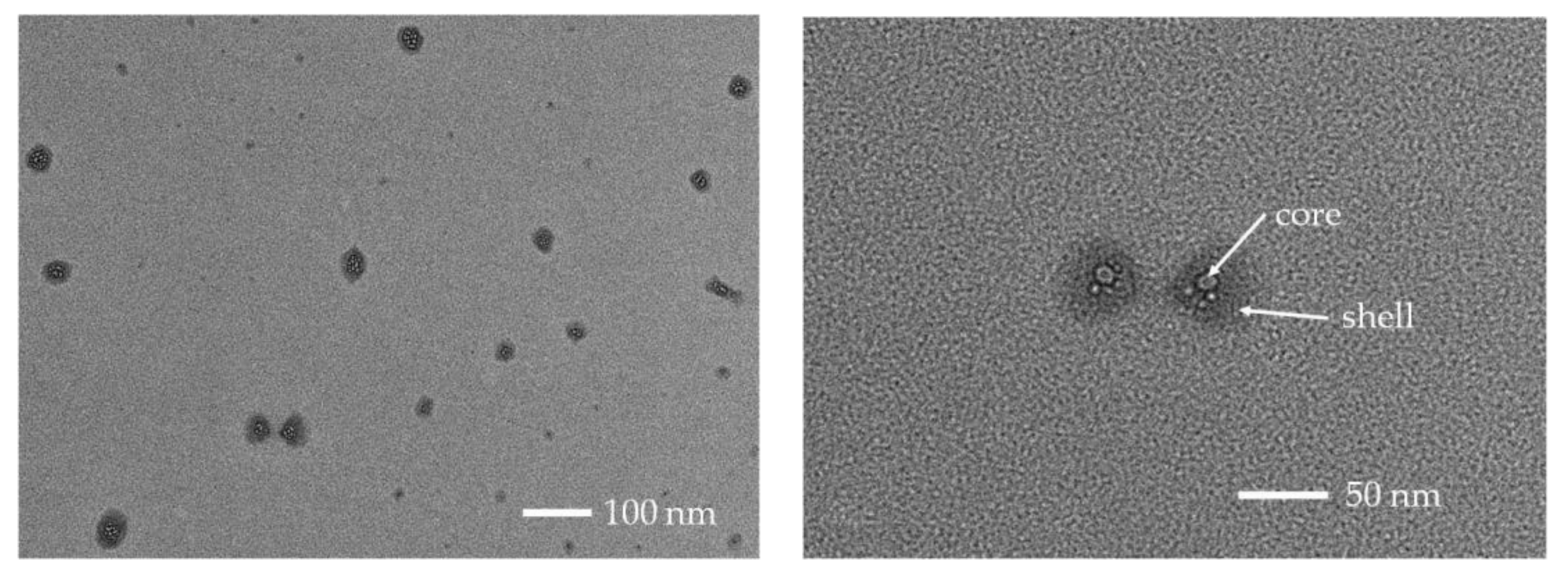
Transmission electron microscopy (TEM) of drug-loaded Ad-(PC_28_-*b*-PD_25_-*b*-PP_8_)_4_ micellles.

As shown in the TEM images (Figure 6), the representative micrograph of drug-loaded Ad-(PC_28_-*b*-PD_25_-*b*-PP_8_)_4_ micelles is presented. The TEM images obviously reveals that the micelles are core-shell structure and filled with DOX. On the other hand, the drug-loaded micelles are about 50 nm spherical particles, which is not in accordance with the data from DLS. This is due to the fact that the TEM images are obtained without the solvent, while the DLS is carried out in solution, and intermicellar aggregation may exist in micellar solution [34,35].

**Figure 7:**
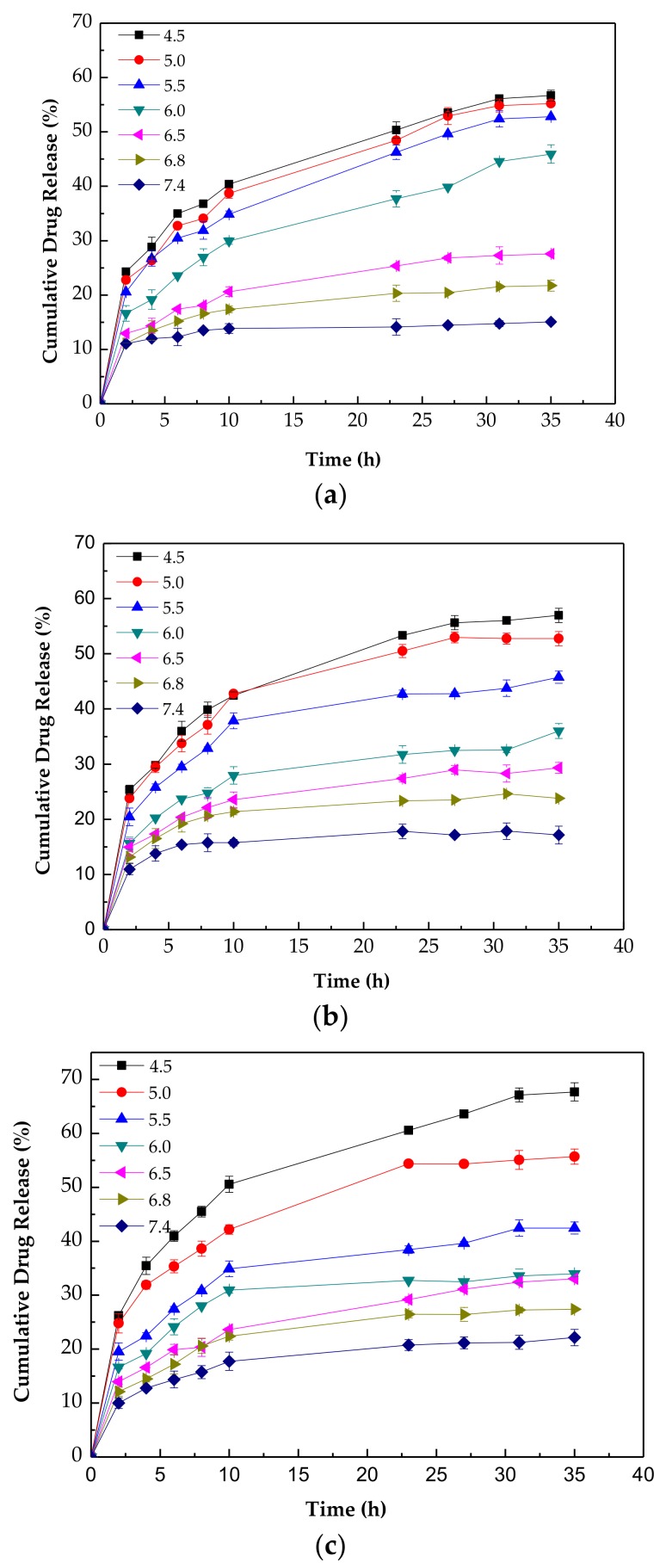
Drug release profiles of DOX from DOX-loaded Ad-(PC_22_-*b*-PD_18_-*b*-PP_8_)_4_ micelles (**a**), Ad-(PC_28_-*b*-PD_18_-*b*-PP_8_)_4_ micelles (**b**), Ad-(PC_28_-*b*-PD_25_-*b*-PP_8_)_4_ micelles (**c**).

In order to investigate the pH-sensitive drug release behavior of micelles, the three drug-loaded Ad-(PCL-*b*-PDEAEMA-*b*-PPEGMA)_4_ micelles were incubated under various pH conditions, and the drug release behavior were monitored using UV–Vis analyzer (Figure 7). When the pH is 7.4, Ad-(PCL-*b*-PDEAEMA-*b*-PPEGMA)_4_ micelles are tight and the release rates of drug are slow, and only 15–22% of DOX is released by 35 h. The release rate of DOX increases slightly as the pH decreases from 6.8 to 4.5, resulting from the partial protonation of the tertiary amine of PDEAEMA. However, 56–67% of DOX is rapidly released at pH 4.5 within the same period, which contributes to the stronger protonation of tertiary amine of PDEAEMA. Furthermore, higher content of the PDEAEMA segment results in greater environmental response performance. At pH = 4.5, 67% of DOX is released from Ad-(PC_28_-*b*-PD_25_-*b*-PP_8_)_4_ by 35 h, which is faster than that of Ad-(PC_28_-*b*-PD_18_-*b*-PP_8_)_4_ (56%). From this point of view, Ad-(PCL-*b*-PDEAEMA-*b*-PPEGMA)_4_ micelles not only can avoid drug bursts during circulation but also enhance intracellular drug release, which is beneficial for controlled drug delivery.

## 4. Conclusions 

In summary, novel star amphiphilic polymers Ad-(PCL-*b*-PDEAEMA-*b*-PPEGMA)_4_ are developed and have been shown to possess low CMC values (0.0025–0.0034 mg/mL), indicating that the adamantane-based polymeric micelles possess favorable stability in solution. Because of unique three-dimensional configuration of adamantane and hydrophobic interaction among the hydrophobic block and DOX, the self-assembled Ad-(PC_28_-*b*-PD_25_-*b*-PP_8_)_4_ micelles exhibit high drug loading capacity (as high as 22.4%). Owing to the protonation of amine groups of PDEAEMA, particle sizes and zeta potentials of micelles show a desired pH-sensitivity, and the release of DOX is accelerated as pH decreases from pH 7.4 (15–22%) to pH 4.5 (56–67%). Overall, this study presents an innovative attempt to introduce the adamantane to improve the drug loading capacity and stability of micelles, and the results indicate that Ad-(PCL-*b*-PDEAEMA-*b*-PPEGMA)_4_ micelles possess a great potential as a vector for controlled delivery of anti-cancer drugs.

## Figures and Tables

**Table 1:** Synthesis Results of Copolymers Ad-(PCL-b-PDEAEMA-b-PPEGMA)_4_.

Sample ^1^	*M*_n,th_^2^	*M*_n,GPC_^3^	*M*_w_/*M*_n_^3^	*M*_n,NMR_^4^
Ad-(PC_22_-*b*-PD_18_-*b*-PP_8_)_4_	40,495	34,524	1.37	36,209
Ad-(PC_28_-*b*-PD_18_-*b*-PP_8_)_4_	43,234	36,735	1.42	38,823
Ad-(PC_28_-*b*-PD_25_-*b*-PP_8_)_4_	48,422	41,793	1.46	44,826

^1^ PC = PCL; PD = PDEAEMA; PP = PPEGMA. the subscripts are the target degree of polymerization of *ε*-CL, DEAEMA and PEGMA. ^2^ Calculated by theory analysis from the feed ratio of *ε*-CL, DEAEMA and PEGMA to initiator. ^3^ Determined by gel permeation chromatography (GPC) with tetrahydrofuran (THF) as the eluent (1.0 mL/min). ^4^ Calculated by the equations *M*_n_,_NMR_ = (114 × *x* + 185 × *y* + 500 × *z*) + 1112. The *x*, *y* and *z* are the degree of polymerization of ε-CL, DEAEMA and PEGMA calculated from ^1^H NMR spectra.

**Table 2:** Particle size, drug loading content (DLC) and entrapment efficiency (EE) of DOX-loaded Ad-(PCL-*b*-PDEAEMA-*b*-PPEGMA)_4_ micelles.

Micelle	DOX/Polymer	Size (nm)	DLC (%)	EE (%)
Ad-(PC_22_-*b*-PD_18_-*b*-PP_8_)_4_	10/40	110.8	7.7	37.8
	20/40	122.5	10.5	31.6
Ad-(PC_28_-*b*-PD_18_-*b*-PP_8_)_4_	10/40	129.0	9.9	40.4
	20/40	152.5	16.7	34.7
Ad-(PC_28_-*b*-PD_25_-*b*-PP_8_)_4_	10/40	180.7	11.6	56
	20/40	256.0	22.4	44.7
